# IP3R deficit underlies loss of salivary fluid secretion in Sjögren’s Syndrome

**DOI:** 10.1038/srep13953

**Published:** 2015-09-14

**Authors:** Leyla Y. Teos, Yu Zhang, Ana P. Cotrim, William Swaim, Jon H. Won, Julian Ambrus, Long Shen, Lolita Bebris, Margaret Grisius, Shyh-Ing Jang, David I. Yule, Indu S. Ambudkar, Ilias Alevizos

**Affiliations:** 1 Sjögren’s Syndrome and Salivary Gland Dysfunction Unit MPTB, NIDCR, NIH, Bethesda, MD 20892; 2 Secretory and Physiology Section, MPTB, NIDCR, NIH, Bethesda, MD 20892; 3 Department of Pharmacology and Physiology, University of Rochester, Rochester, NY 14642; 4 Division of Allergy, Immunology and Rheumatology, Department of Medicine, School of Medicine and Biomedical Sciences, State University of New York at Buffalo, Buffalo, NY 14203.

## Abstract

The autoimmune exocrinopathy, Sjögren’s syndrome (SS), is associated with secretory defects in patients, including individuals with mild lymphocytic infiltration and minimal glandular damage. The mechanism(s) underlying the secretory dysfunction is not known. We have used minor salivary gland biopsies from SS patients and healthy individuals to assess acinar cell function in morphologically intact glandular areas. We report that agonist-regulated intracellular Ca^2+^ release, critically required for Ca^2+^ entry and fluid secretion, is defective in acini from SS patients. Importantly, these acini displayed reduction in IP3R2 and IP3R3, but not AQP5 or STIM1. Similar decreases in IP3R and carbachol (CCh)-stimulated [Ca^2+^]_i_ elevation were detected in acinar cells from lymphotoxin-alpha (LTα) transgenic (TG) mice, a model for (SS). Treatment of salivary glands from healthy individuals with LT α, a cytokine linked to disease progression in SS and IL14α mice, reduced Ca^2+^ signaling. Together, our findings reveal novel IP3R deficits in acinar cells that underlie secretory dysfunction in SS patients.

Primary Sjögren’s syndrome (pSS) is a chronic autoimmune disease involving lymphocytic infiltration and loss of secretory function in salivary and lacrimal glands^1^. Loss of salivary fluid secretion results in xerostomia, which leads to complications such as difficulty swallowing, rampant dental caries, oral mucosal lesions, and fungal infections that together severely affect the quality of life for the patients^2^. In addition, SS also presents with extra-glandular systemic manifestations that may impact tissues such as the skin, heart, lungs, kidney, gastrointestinal and endocrine system, as well as the central and peripheral nervous system^3^. The current criteria used for the diagnosis of pSS include: subjective and objective signs of dry mouth and/or dry eyes, the presence of anti-Ro/anti-La autoantibodies, and histological evaluation of the minor salivary glands for lymphocytic infiltration^4^. The onset and progression of the disease, as well as severity of inflammation and loss of secretory function varies between the two exocrine glands and even within the different salivary glands. For example, in most patients submandibular and minor salivary glands appear to be impacted first followed by the parotid gland. Sublingual glands are most often not affected. Involvement of lacrimal glands may occur before, along with, or independent of the loss in salivary gland function. While the pathogenesis of this disease has not yet been elucidated, it has been suggested that viral, hormonal, genetic, environmental, and neurophysiological factors might contribute to the initiation and progression of the disease^5^,^6^,^7^.

A major and unresolved conundrum in the pathophysiology of (SS) has been the lack of correlation between salivary flow and extent of inflammation or tissue damage^6^,^8^. This is very relevant in the case of patients who display low levels of inflammation within their salivary glands (i.e. a relatively large part of the gland is histologically intact with little overt damage) and yet have substantial loss of function. It has been suggested that this might represent an early stage of the disease, although some patients do not progress to the more severe disease state.

Efforts to delineate the initial molecular alterations that underlie the secretory defect have been complicated by the fact that pSS is a slowly progressing disease, the early stages of which have proved difficult to identify. There are relatively few animal models that recapitulate the phenotype of disease progression, especially loss of saliva flow in absence of significant inflammation and glandular damage^9^. A number of these focus on elucidating the potential role of B cells in the pathogenesis of (SS). These include the mice expressing B cell activating factor (BAFF), Act1−/− mice (lacking Act1 a negative regulator of BAFF and CD40), as well as the more widely used NOD mice. While studies with these mice support the association of B cell activation during onset of disease and hypofunction of salivary glands, none of them recapitulate primary aspects of the disease, including its slow progression. The IL14α (TG) mice display many features of (SS)^10^,^11^,^12^,^13^. In particular, the onset and slow progression of the disease as well as timing of specific exocrine gland involvement are similar to pSS in humans. Furthermore, as in patients, IL14α (TG) mouse demonstrates an increase in autoantibodies and cytokines first followed by lymphocyte infiltration of salivary and lacrimal glands and finally glandular destruction. Notably, loss of fluid secretion is seen in the absence of extensive lymphocytic infiltration of salivary glands or tissue damage, a condition very similar to that described above in pSS patients. The onset and progression of the disease in the IL14α (TG) mouse has been linked to an elevation of (LTα), a member of the Tumor Necrosis Factor (TNF) family of proteins. Notably, pSS-like symptoms were not seen in IL14α (TG) mice that lack LTα^11^. On the other hand, the more delayed manifestations of the disease in the mice, including systemic inflammation and lymphoma, appear to be driven by Interferon-α (IFNα) as these features are absent in the IL14α (TG) animals lacking type 1 interferon receptor. Notably, both LTα and IFNα levels are increased in saliva and serum of pSS patients. Consistent with this, LTα is also upregulated in patients with autoimmune pancreatitis and mice with targeted expression of the cytokine in pancreatic acinar cells induced the autoimmune disorder^14^. Thus, it has been suggested that LTα may play a prominent role in the early loss of salivary function in pSS and in progression of the disease^11^. Little is known regarding the mechanisms causing the early loss of saliva secretion in patients and animal models. The current studies were directed towards addressing this issue.

Salivary glands mediate vectorial fluid secretion in response to stimulation by neurotransmitters. The key triggering event is neurotransmitter induced increase in cytosolic [Ca^2+^] ([Ca^2+^]_i_) in acinar cells, the primary site of secretion in the gland, which leads to activation of ion channels and transporters which together generate the osmotic gradient required to drive water from the cell^15^,^16^,^17^. Physiologically, the increase in [Ca^2+^]_i_ is achieved as a result of intracellular Ca^2+^ release from the endoplasmic reticulum as well as Ca^2+^ entry into the acinar cells, both of which are activated in response to stimulation of the plasma membrane receptors, such as muscarinic or alpha-adrenergic receptors. Such stimuli result in hydrolysis of phosphatidylinositol 4,5 bisphosphate (PIP2) and generation of inositol 1, 4, 5 trisphosphate (IP3)^18^. IP3 binds and activates IP3 receptors (IP3R) located in the endoplasmic reticulum (ER) resulting in the release of Ca^2+^ from the ER lumen^19^. The resulting elevation in cytosolic Ca^2+^ [Ca^2+^]_i_ tends to be transient in the absence of extracellular Ca^2+^. A sustained elevation of [Ca^2+^]_i_, which is critical for prolonged saliva flow, requires Ca^2+^ entry,^20^,^21^. Ca^2+^ entry in acinar cells, the site of water movement in the gland, is primarily mediated via the store-operated Ca^2+^ entry (SOCE) mechanism, which is activated by the decrease in ER-[Ca^2+^]. SOCE is regulated by the ER protein, STIM1 (stromal interaction molecule 1), which senses the decrease in ER-[Ca^2+^] and interacts with plasma membrane calcium channels, Orai1 and TRPC1, resulting in their activation. Ca^2+^ entry via these channels is required for maintaining the increase in [Ca^2+^]_i_ critical for the regulation of fluid secretion^22^,^23^. Essentially, the increase in [Ca^2+^]_i_ activates key K^+^ and Cl^-^ channels that together generate the osmotic gradient required to drive fluid secretion via the water channel, AQP5. Thus, intracellular Ca^2+^ release, via IP3R, in acinar cells represents the primary, critical step in neurotransmitter-regulation of fluid secretion.

We hypothesized that functional defects in acinar cells, the primary site of water secretion in the salivary glands, could account for the secretory dysfunction in patients with minimum lymphocytic infiltration and histologically detectable damage in salivary glands. To test this hypothesis, we utilized live cell imaging with acinar lobule preparations of minor salivary gland biopsies from pSS patients and healthy volunteers (HV). Specifically, the technique allowed us to select acinar cells in areas of the gland that were relatively intact with normal morphology. Our findings demonstrate that critical processes involved in fluid secretion are disrupted in acinar cells from SS patients; namely intracellular Ca^2+^ release, Ca^2+^ entry, and cell volume reduction. Importantly, we show that IP3Rs, but not AQP5, are reduced in salivary gland acinar of pSS patients, which can account for the aberrant fluid secretion in the patients. These findings were further validated using the IL14α-TG mice model since it most closely resembles the development of SS in humans. Submandibular gland acinar cells from IL14α-TG mice display a similar deficiency in IP3R accompanied by reductions in muscarinic receptor-regulated Ca^2+^ signaling. Finally, we show that incubation of minor salivary gland biopsies from healthy patients with LTα induce attenuation of muscarinic receptor-stimulated Ca^2+^ signaling. Together our findings provide evidence that deficits in IP_3_Rs underlie the secretory defect in pSS. Further, our data indicate that this defect might be associated with an ambient increase in LTα within the gland, possibly in the early stages of the disease. We propose that strategies aimed towards blocking the cytokine, or effects thereof, early in the disease process, will be potentially useful in preventing loss of salivary function and treatment of pSS.

## Results

### Loss of saliva secretion in pSS patients displaying mild inflammation in salivary glands

**Figure 1:**
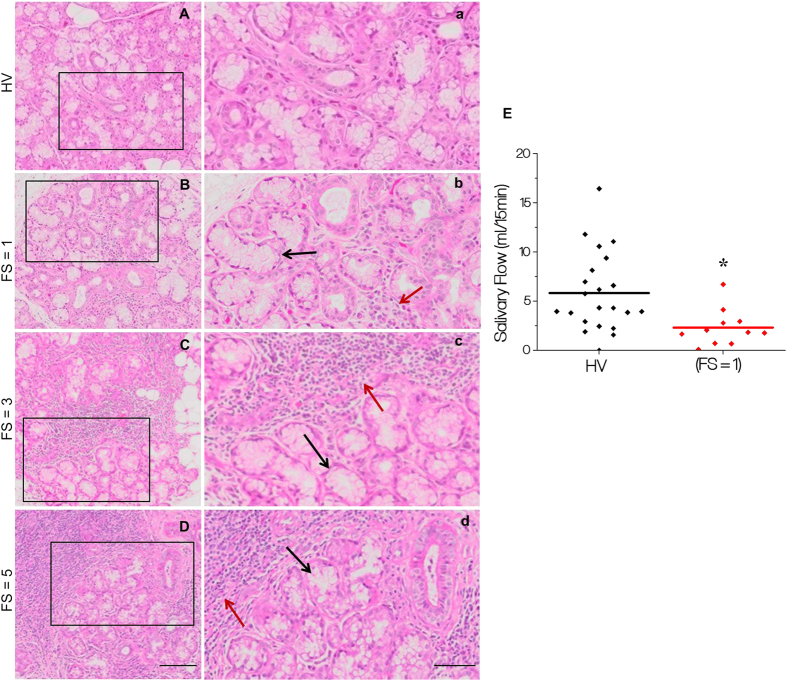
Morphology of minor salivary glands from healthy volunteers (HV) and primary Sjögren’s patients (pSS). Samples from (HV) and pSS patients with focus scores (FS) of 1, 3 and 5 were stained with hematoxolin and eosin. Morphology was assessed using 20× objectives. (**A–D** show larger areas of the tissue sample while enlarged areas of the portions marked are shown in (a–d). Relatively intact morphologically normal looking areas in the sections are shown by black arrows, while infiltrated areas are indicated by red arrows. Samples from pSS patient with FS = 5 (**D**, d) displays diffused infiltration with little residual gland morphology (**E**) Displaying whole unstimulated salivary flow measured in individual HV (N = 22) and pSS FS = 1 patients (N = 11). Mean ± SEM for the HV group is 5.827 ± 0.8556 ml/15 min and for the pSS group, 1.759 ± 0.4334 ml/15 mins (* denotes a significant difference between Means, p < 0.0001). (**A–D**, scale bar = 100 microns) (a–d, scale bar = 50 microns).

The morphology of minor salivary gland biopsies from healthy volunteers (HV) and pSS patients, with varying focus scores (FS) between 1–5 (based on scaling between 0–12), was examined by hematoxylin and eosin staining of tissue sections obtained from the biopsies. HV glands demonstrated normal morphology without any detectable inflammation (Fig. 1A and a). Samples from pSS patients with FS = 1, FS = 3, and FS = 5 displayed increasing infiltration, with more foci as well as spread of infiltrate within each foci (marked by red arrows in Fig. 1b–d). Notably, areas away from the site of infiltration appeared to be morphologically intact and normal. As expected, such areas were relatively more frequent in samples from FS = 1 patients (marked by black arrows). In all cases, there was distinct loss of salivary gland structure within the area of infiltration (indicated by red arrows in the images). Importantly, despite the presence of relatively large areas of intact tissue in the gland, patients with FS = 1, demonstrated significant loss (Fig. 1E) of salivary secretion as compared to HV. Details of clinical features of the patients are described in Supplemental Table 1. In the following sections, we describe detailed functional analysis of acinar cells within the relatively normal appearing areas of glands from pSS patients with low focus score.

### Agonist-induced decrease in cell volume is attenuated in acinar cells from pSS patients

**Figure 2:**
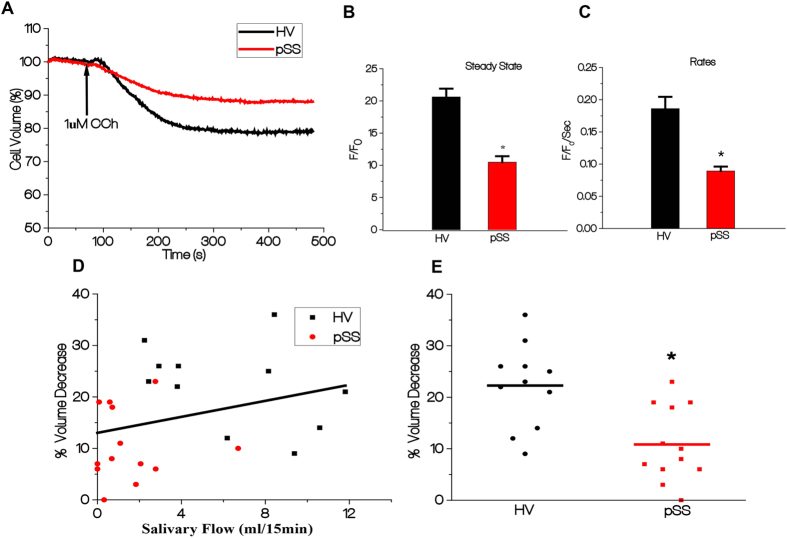
Carbachol-stimulated decrease in acinar cell volume is attenuated in minor salivary gland biopsies from pSS patients. (**A**) Calcein fluorescence was monitored in acinar cells using confocal microscopy. The data in A were used to quantitate: (**B**) Decrease in volume in HV (20.61017 F/F_0_ ± 1.29369) vs in the pSS patients (10.4703 F/F_0_ ± 0.94543) (**C**) Initial rate of volume decrease in HV and pSS cells (0.1861 F/F_0_/Sec ± 0.01837 in the HV versus 0.09333 F/F_0_/Sec ± 0.006863 in the pSS individuals (*p *< 0.0001). (**D**) Spearman r correlation between CCh-stimulated volume decrease and salivary flow in the pSS patients (red symbols) and HV (black symbols) (*p *= 0.0323, eleven HV, and thirteen pSS). (**E**) Spread of CCh-stimulate volume changes in individual patients from HV and pSS groups (*p *= 0.0010). * indicates significant difference.

We first examined CCh-stimulated decrease in cell volume in acinar cells, a readout of fluid secretion, using lobule preparations of lower labial minor salivary gland biopsies from HV and pSS patients (see Methods for details). Only intact acinar cells accumulate and retain fluorescent dyes which can be microscopically selected for measurements^24^,^25^. Thus, this technique allowed us to measure the function of intact acini in pSS patient glands. Stimulation of cell lobules with relatively low, submaximal, [CCh] (1 μM CCh) induced a 20% decrease in volume (which reached steady state at about 300 sec) of acinar cells from HV. CCh-stimulated decrease in cell volume, about 10%, was significantly less in acinar cells of pSS glands compared to that in HV acini (Fig. 2B). A representative trace is shown (Fig. 2A) from one experiment (single biopsy) using data obtained from 3–4 samples (average of minimum 40 ROI). Steady-state values of cell volume 300 sec after stimulation with 1μM carbachol (CCh) was HV = 79.39% ± 1.294, (n = 80 cells, N = 11 patients) and in pSS cells 89.53% ± 0.9454, (n = 78 cells, N = 13 patients) (p = 0.0019). The initial rate of cell volume decrease was also significantly different in pSS acini compared to that of HV acini. These data suggest that acinar cells in minor salivary glands of pSS patients with low focus scores, have a secretory defect. Importantly, there was a significant correlation between agonist–induced volume decrease in acinar cells and salivary secretion of the patients (Fig. 2D, HV, black symbols, and pSS patients, red symbols, Spearman r = 0.4380; *p *= 0.0323). CCh-stimulated volume decrease in acinar cells of individual patients within each group is shown in Fig. 2E. Together, these data demonstrate that agonist-stimulated acinar cell volume decrease appears to be an accurate indicator of the functional status (salivary gland fluid secretion) of the patients.

### Carbachol-stimulated [Ca^2+^]_i_ mobilization is reduced in minor salivary gland acinar cells from SS patients

**Figure 3:**
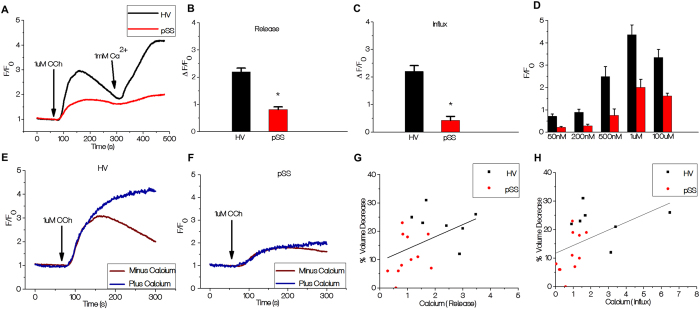
Carbachol-stimulated Ca^2+^ signaling is decreased in pSS. (**A**) Fluo2 fluorescence was recorded in cell lobules and stimulated with 1μM carbachol in Ca^2+^ free medium, followed by inclusion of 1 mM Ca^2+^ to the external medium. Traces are representative of data from one experiment, values are averages from 3-4 samples (minimum of 40 ROIs) (**B**) Quantitation of first peak increase in fluorescence (representing internal [Ca^2+^]_i_ release in cells from pSS patients (0.8129 ± 0.09980) in 121 cells from eleven patients) and seven HV individuals (2.196 F/F_0_ ± 0.1357, 105 cells, 7 patients), * indicates significant difference (P < 0.0001). (**C**) Quantitation of second peak of fluorescence increase (due to Ca^2+^ entry) from eleven pSS patients and seven HV (0.4275 ± 0.14583 121 cells and 2.20134 F/F_0_ ± 0.2099, 105 cells). * indicates a significant difference P<0.0001. (**D**) Dose-dependence of CCh-stimulated increases in [Ca^2+^]_i_ in acinar cells from HV (5 individuals) and pSS patients glands (8 patients) in Ca^2+^-containing medium. (E&F) CCh-stimulated increase in fluorescence in Ca^2+^-containing and Ca^2+^-free media from HV and pSS patients. (G&H) Spearman r correlation between CCh-stimulated volume changes versus intracellular Ca^2+^ release (*p *= 0.0166) and Ca^2+^ influx (*p *= 0.0043), respectively, in seven HV and eleven pSS patients.

The primary determinant of cell volume decrease in response to agonist stimulation is an increase in cytosolic calcium. To determine whether defects in Ca^2+^ signaling can account for the decrease in cell volume change, we assessed CCh-induced [Ca^2+^]_i_ increases in acinar cells by measuring changes in Fluo2 fluorescence in lobule preparations from seven HV and eleven pSS patients. Agonist-stimulated intracellular Ca^2+^ release was initiated by stimulating cells with 1 μM CCh in the absence of extracellular calcium, measured as a transient increase in fluorescence. Subsequent addition of 1mM calcium to the external medium triggers a second, more sustained, increase in fluorescence due to Ca^2+^ entry. Figure 3A displays the pattern of [Ca^2+^]_i_ changes in acinar cells from pSS patients and HV showing the calcium release and entry phases. CCh-stimulated intracellular Ca^2+^ release was significantly reduced (>50%, p < 0.0001) in pSS acini compared to that in HV acini (Fig. 3B). Cells from pSS patients also showed significant reduction (about 75%) in the Ca^2+^ entry component as compared to that in HV cells (Fig. 2C). Since CCh-stimulated Ca^2+^ entry in acinar cells is mediated primarily via the store-operated Ca^2+^ entry pathway (SOCE)^26^, the reduction in Ca^2+^ entry detected in the pSS patients is most likely due to a reduction in intracellular Ca^2+^ release. Thus, while we cannot exclude that conditions associated with pSS might directly affect mechanism and components involved Ca^2+^ entry, we have not examined Ca^2+^ entry in further detail.

[Ca^2+^]_i_ increase in response to varying [CCh], ranging from 50 nM to 100 μM, was measured in eight pSS patients and five HV. Cells from pSS patients showed a significant reduction (about 50%) in [Ca^2+^]_i_ increase at all [CCh] tested compared to the responses in cells from HV (Fig. 3D). This indicates an overall decrease in CCh-induced Ca^2+^ mobilization rather an alteration in the sensitivity of the cells to CCh. We further examined CCh-induced [Ca^2+^]_i_ increases in cells maintained in a Ca^2+^-containing medium. Cells from both HV and pSS patients showed an initial increase in [Ca^2+^]_i_ that was not affected by inclusion of Ca^2+^ in the external medium (Fig. 3E,F) and a subsequent more sustained elevation seen only in cells bathed in Ca^2+^-containing external medium. The initial Ca^2+^ increase was slower in cells from pSS patients, in agreement with slower intracellular Ca^2+^ release. Importantly, there was a significant correlation between CCh-induced decrease in cell volume and Ca^2+^ release (Spearman r = 0.5561) (*p *= 0.0166) as well as Ca^2+^ influx (Spearman r = 0.6388) (*p *= 0.0043) (Fig. 3G,H). Again, as seen in the case of cell volume measurements, there was a clear separation between the values obtained in HV and pSS patients. Further, there was a significant correlation between patient saliva flow and Ca^2+^ release (Spearman r = 0.5741) (*p *= 0.0127) as well as calcium influx (Spearman r = 0.5844) (*p *= 0.0109) (Supplemental Fig. 4). Since CCh-stimulated Ca^2+^ increases are correlated with volume changes in acinar cells as well as saliva secretion in patients, we suggest that the defect in CCh-stimulated intracellular Ca^2+^ release detected in acini from pSS patients, can account for the reduced salivary secretion in pSS patients with low focus scores who display minimal damage of salivary gland tissue.

### IP3R2 and IP3R3 are decreased in all areas within salivary glands from SS patients

**Figure 4:**
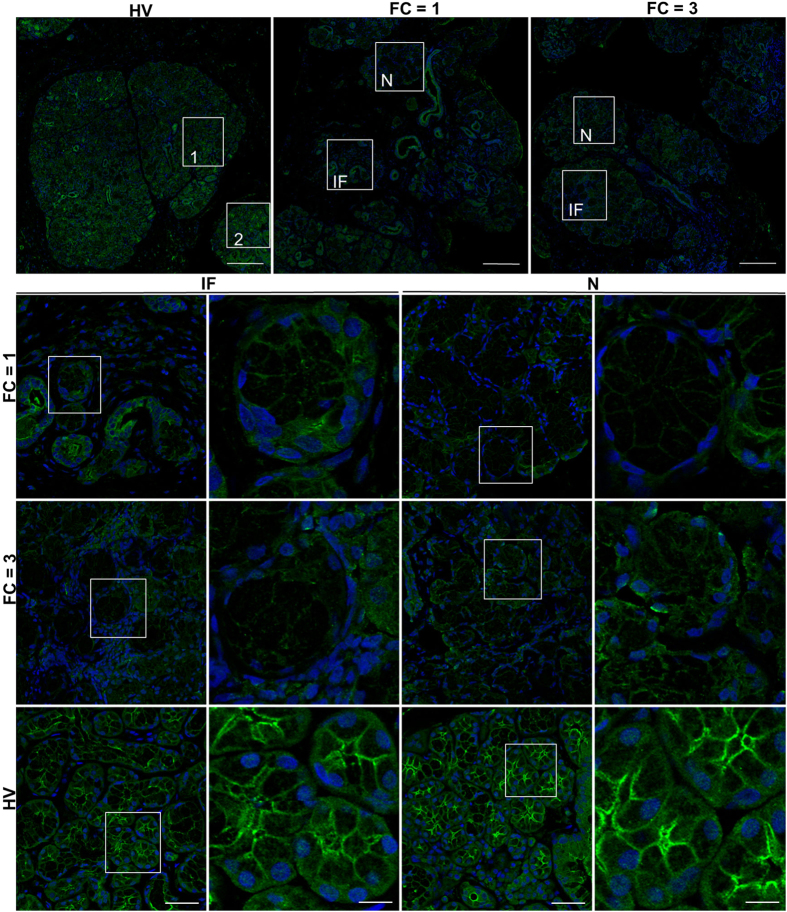
IP3R3 expression in minor salivary glands biopsies of pSS patients and healthy volunteers. Stitched Immunofluorescence images of IP3R3 in entire biopsied sample area (scale bar = 300 microns). Area labeled 1 and 2 were randomly picked areas from HV. Representative images of IP3R3 detected by immunofluorescence in salivary gland sections from pSS patients with FS = 1, FS = 3. An area from within the infiltration (IF) was picked and an area away from infiltration where tissue appeared to be morphologically intact (N). In each case, enlarged images (of areas marked by white boxes) are shown in the second panel to the right (scale bar = 20 microns). Areas were picked from stitched images shown in the 1^st^ panel (scale bar = 50 microns).

**Figure 5:**
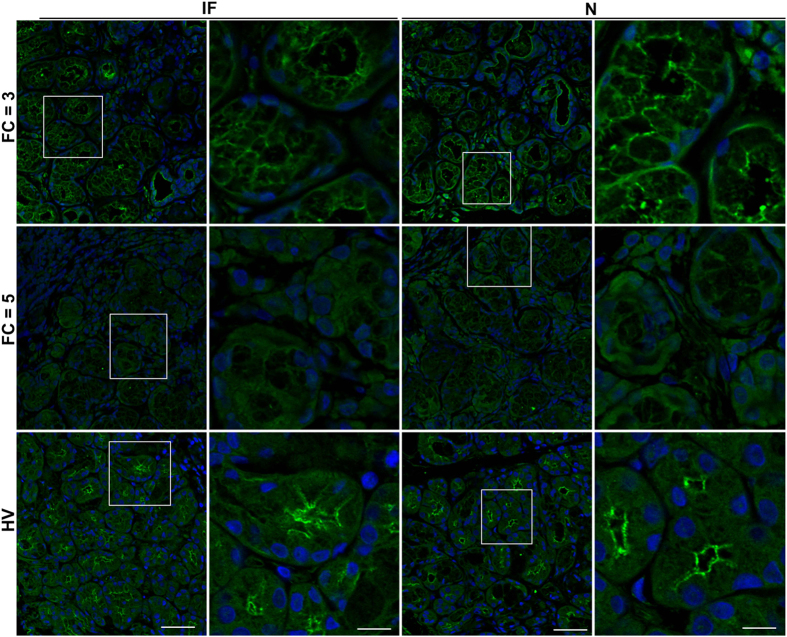
IP3R2 expression in minor salivary glands biopsies of pSS patients and healthy volunteers. Representative images of IP3R2 detected by immunofluorescence in salivary gland sections from pSS patients with FS = 3, FS = 5, and healthy volunteers (HV) (scale bar = 50 microns). An area from within the infiltration (IF) was picked and an area away from infiltration where tissue appeared to be morphologically intact (N). In each case, enlarged images (of areas marked by white boxes) are shown in the second panel to the right (scale bar = 20 microns).

IP3R2 and IP3R3 are critical for mediating intracellular Ca^2+^ release in salivary gland acinar cells. Lack of these receptors in mice led to a complete loss of fluid secretion^27^. We assessed the expression and localization of IP3R3 (Fig. 4) and IP3R2 (Fig. 5) in salivary gland tissue samples from HV and pSS patients with FS = 1 and FS = 3 for IP3R3 and FS = 3 and FS = 5 for IP3R2. AQP5, the major water channel in salivary glands and STIM1, the protein regulating SOCE, were also examined. Representative stitched images for IP3R3 (Fig. 4), and for STIM1, and AQP5 across entire sections of the glands from the same patient are shown (Supplemental Fig. 2). Marked areas in each image represent an area of infiltration (IF) or one that is away from the site of infiltration where tissue appears to have normal morphology (N). In HV samples, STIM1 labeling was clearly detected within the acinar cell area while strong IP3R3 and AQP5 signals were detected within the apical region of the acinar cells, as previously reported^28^,^29^,^30^,^31^. All three proteins were uniformly detected across the entire section of the gland. In the case of samples from pSS patients, all three proteins were poorly detected within the IF region, in FS = 1 and FS = 3 patient samples (Fig. 4, Supplemental Figures 2, 3, and 4). Importantly, while AQP5 and STIM1 were readily detected in areas away from the infiltrating sites, IP3R3 signal was low in a major portion of the tissue sections from FS = 1 and FS = 3 pSS patients. High resolution imaging demonstrated very weak labeling within the apical region of acini in both IF and N areas in pSS glands (Fig. 4, shows enlarged images of the marked areas well as enlarged images of acinar cells from HV and N of FS = 1). We also examined presence and localization of IP3R2 which, like IP3R3, was strongly detected at the apical region in acinar cells in glands from HV (Fig. 5). However, in FS = 3 and FS = 5 patients, the signal was reduced within infiltrating areas (IF), with greater overall reduction in FS = 5 patients. Importantly, in FS = 5 patients acinar cells within the relatively intact areas (N) displayed mislocalization of IP3R2 (Fig. 5).

In aggregate, these findings demonstrate that STIM1 and AQP5 are reduced within the area of infiltration (IF) in glands from pSS patients, but not in areas away from the infiltration site. Thus, loss of these proteins can only contribute to loss of Ca^2+^ entry and fluid secretion function within site of infiltration. Importantly, our findings reveal a novel deficit in IP3Rs in pSS patients that is detected in acinar cells within the relatively normal areas of the pSS gland that are away from the site of infiltration. Although we cannot completely rule out effects due to other possible contributing factors, the present findings strongly suggest that the decrease in IP3Rs in acinar cells can be the major underlying cause for the decrease in salivary flow in patients with low focus scores.

### Attenuation of acinar cell Ca^2+^ Signaling and IP3R in IL14α transgenic mice

**Figure 6:**
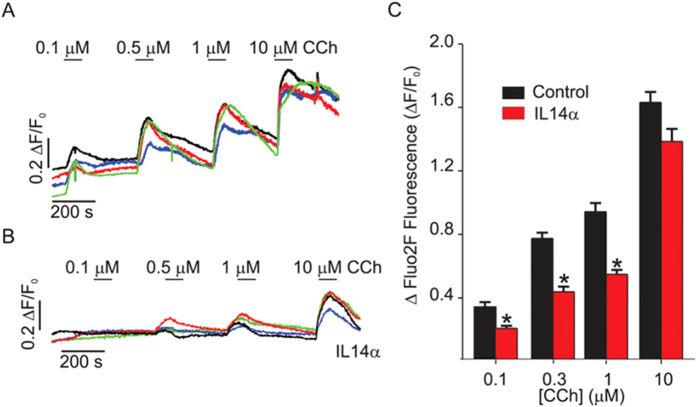
Ca^2+^ Signaling in lobules from IL14 alpha transgenic mice. (**A**) Representative traces from female, age matched wild type controls showing the concentration-dependent change in [Ca^2+^]_i_ stimulated by a range of [CCh] in submandibular acini. The traces represent the normalized change in fluorescence for an individual cell within an acinus. Cells from different acini in the field of view in one lobule are presented. Experiments were performed on at least 9 lobules from 3 different animals. (**B**) [Ca^2+^] signals are significantly reduced in IL14α animals. (**C**) Pooled data. [Ca^2+^] signals are significantly reduced in response to sub-maximal [CCh] *p = <0.01.

The IL14α-TG mice has been reported to faithfully reproduce many of the features of primary SS including its relatively slow and chronic progression^12^. Previous studies have shown that stimulated fluid secretion is attenuated in submandibular glands at 10 months of age in IL14α-TG mice. Importantly, in common with low focus score patients, loss of fluid secretion is not associated with significant lymphocyte infiltration or glandular damage. We have investigated submandibular gland acinar cells from this mouse to determine whether they display characteristics similar to those observed in human pSS minor salivary glands. [Ca^2+^]_i_ measurements were performed in fluo2-loaded lobules isolated from 10 month old IL14α-TG mice or age, sex and genetic background matched control animals as described in Materials and Methods. The magnitude of the CCh-induced peak in [Ca^2+^]_i_ measured in lobules isolated from female mice were markedly reduced at sub-maximal stimulation (Fig. 6). Additionally, intracellular Ca^2+^-store status was assessed by treating submandibular gland acinar cells from the two sets of mice with CPA in Ca^2+^-free external medium. There was no difference in the overall pattern of [Ca^2+^]_i_ increase in the two sets of cells; values obtained for peak [Ca^2+^]_i_ increase, time to reach half peak height, as well as area under the curve were all similar (Supplementary Figure 5). These data rule out the possibility that defects in intracellular Ca^2+^ store content can account for CCh-mediated intracellular [Ca^2+^]_i_ increase. Notably, Ca^2+^ signaling in submandibular glands from male IL14α-TG mice was largely unaltered, consistent with the female: male predominance of pSS in humans.

**Figure 7:**
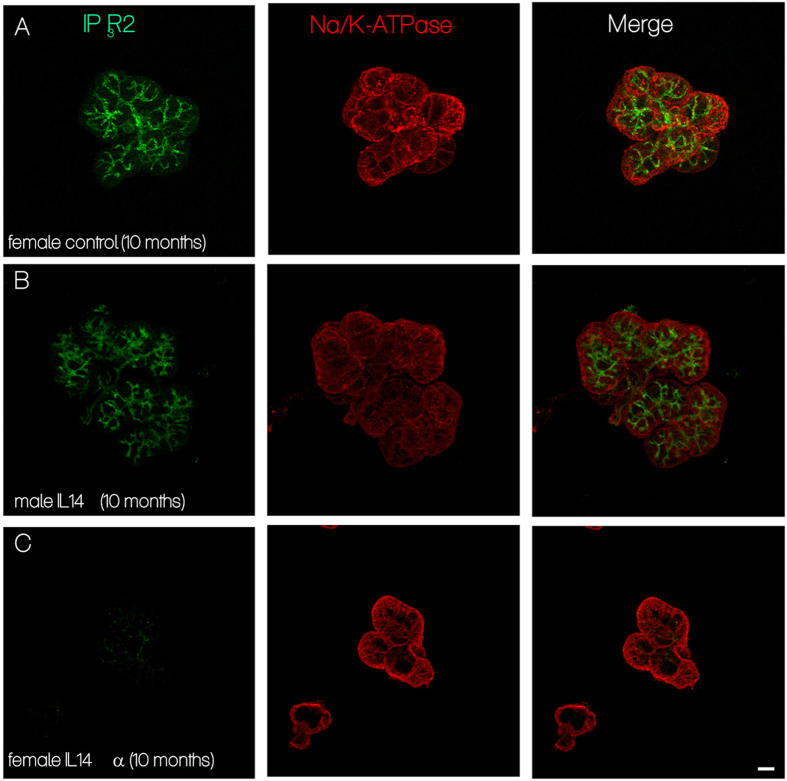
IP3R2 expression in submandibular salivary glands from female IL14 alpha transgenic mice. (**A)** Left panel shows the typical apical staining of IP3R2 in age matched control animals. Middle panel show the basal localization of the Na/K-ATPase. Right panel overlay of IP3R2 and Na/K-ATPase localization. (**B**) Left panel shows the typical apical staining of IP3R2 in male 10 month old IL14a animals. Middle panel show the basal localization of the Na/K-ATPase. Right panel overlay of IP3R2 and Na/K-ATPase localization. (**C**) Left panel shows the absence of typical apical staining of IP3R2 in female 10 month old IL14a animals. Middle panel show the basal localization of the Na/K-ATPase. Right panel overlay of IP3R2 and Na/K-ATPase localization. (scale bar = 10 microns).

Next, we examined the subcellular localization of IP3R2 in submandibular glands from IL14α-TG mice and control animals. While the characteristic strong apical localization of IP3R2 could be readily demonstrated in both female age matched control animals and male IL14α (Fig. 7A,B, left panels), IP3R2 was largely absent in female IL14α-TG animals (Fig. 7C), while prominent expression of the basally localized Na/K-ATPase was readily apparent in submandibular tissue samples from all animals (Fig. 7A–C, right panels). These findings are entirely consistent with the data obtained in acinar cells from SS patients, namely that a primary mechanism which underlies the loss of fluid secretion in both the mouse model and patients is an attenuation of Ca^2+^ signaling resulting from a reduction in apically localized IP3R.

### Ca^2+^ signals are attenuated in human minor salivary glands from healthy individuals after treatment with LTα

**Figure 8:**
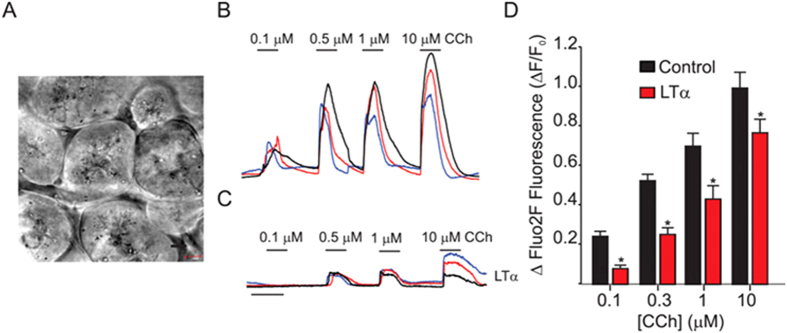
Ca^2+^ signaling in minor salivary glands from HV incubated with (LTα). **(A**) Representative traces from lobules prepared from HV minor salivary glands incubated at 37 °C for 16 hrs showing the concentration-dependent change in [Ca^2+^]_i_ stimulated by a range of (CCh). The traces represent the normalized change in fluorescence for an individual cell within an acinus. Cells from different acini in the field of view in one lobule are presented. Experiments were performed on at least 9 lobules from 4 individual volunteers. (**B**) [Ca^2+^] signals are significantly reduced in lobules incubated with 100 ng/ml (LTα). (C) Pooled data. [Ca^2+^] signals are significantly reduced in response to sub-maximal [CCh] *p = <0.01.

As discussed above, cytokines, in particular, IFNα and LTα, are believed to play a major role in the initiation and progression of Sjögren’s Syndrome^6^,^32^. In IL14α-TG animals, the initiation of SS-like disease is dependent on the expression of LTα^11^, while the later systemic inflammation and development of lymphoma appears driven by IFNα^33^. Thus, in order to investigate whether LTα could play a similar initiating role in the development of Sjögren’s Syndrome in humans we monitored [Ca^2+^]_i_ signals in lobules prepared from minor salivary glands obtained from HV that were incubated with LTα for 16 hrs. Figure 8A shows a DIC image of the lobule from HV, demonstrating that the polarized morphology of the tissue was retained following 16 hour culture. Robust CCh-induced Ca^2+^ signals could be evoked in lobules in vehicle-treated controls (Fig. 8B). Strikingly, the magnitude of [Ca^2+^]_i_ signals were significantly reduced in minor salivary gland lobules following incubation with LTα (Fig. 8C and pooled data in 8D). These data are consistent with a common, early event in pSS being a reduction in Ca^2+^ signaling mediated by signal transduction downstream of increases in LTα which results in loss of glandular fluid secretion.

## Discussion

The molecular mechanism(s) underlying exocrine gland dysfunction and pathogenesis in pSS patients is unknown. While destruction of glandular structure due to widespread lymphocytic infiltration can contribute to the decrease of salivary fluid secretion, it does not explain the loss of secretion in the majority of patients who have low, or negligible, levels of inflammation. Salivary glands in these patients are largely intact, without overt damage to majority of the acinar cells. The data we present here reveal that agonist-stimulated Ca^2+^ signaling is attenuated in acinar cells that are present in the relatively intact areas of gland from patients with low focus scores. Importantly, we have identified a defect in IP3Rs in acinar cells of glands from pSS patients that can account for the loss of Ca^2+^ signaling as well as saliva secretion. Additionally, we show that similar defects in Ca^2+^ signaling and IP3R are present in acinar cells from IL14α-TG mice, an animal model for SS that closely resembles the development of this disease in humans. Increases in LTα, a member of the TNF family, in serum and salivary glands has been associated with early stages of the autoimmune disease in both pSS patients and IL14α-TG mice. Importantly, we show that *in vitro* treatment of salivary glands from healthy patients with LTα also induces loss of Ca^2+^ signaling. Together, our findings suggest that defective Ca^2+^ signaling due to loss of IP3Rs in acinar cells can account for the attenuation of fluid secretion in pSS patients with low levels of lymphocytic infiltration and minimal tissue damage.

Water secretion from acinar cells is completely dependent on agonist-stimulated [Ca^2+^]_i_ increases and IP3R activation is the critical first step in his process, as it also governs the activation of Ca^2+^ entry^26^. We show that while key proteins involved in Ca^2+^ mobilization and fluid secretion; STIM1, and IP3R, and AQP5, are disrupted within, or immediately around the area of infiltration; where there is considerable tissue damage, only IP3Rs are significantly decreased in the relatively intact areas of the gland where there is no infiltration. While we cannot rule out additional defects in components involved in mediating and regulating Ca^2+^ entry, the observed loss of IP3Rs in acinar cells located in relatively intact areas of the gland in patients with low inflammatory scores can account for the decrease in CCh-stimulated intracellular Ca^2+^ release and Ca^2+^ entry, CCh-stimulated reduction in cell volume, and consequently, saliva secretion. Based on previous studies with the IL14α-TG mice and the role of LTα in pSS^11^,^33^, we suggest that the observed loss of IP3R represents a relatively early event in the progress of the disease. Our findings with IL-14α-TG mice, where LTα plays a major role in disease initiation, together with the observation that exposure of normal human tissue to LTα also results in diminished Ca^2+^ signals suggests that important signaling events regulated by LTα might be involved. LTα acts on TNF receptors leading to a multitude of signaling pathways including activation of NFAT, caspases, MAP kinase cascades, and NFκB-mediated transcriptional pathways^34^, many of which are documented to impact IP3R function^35^,^36^. Conceptually, the decrease in IP3R protein could occur by a combination of either a reduction in production, or alternately by an increase in degradation. While there is very little published data relating to transcriptional control of IP3R2 and IP3R3 genes, there are a number of reports documenting the mechanisms that degrade IP3Rs. For example, IP3R2 and IP3R3 in acinar cells are substrates for ubiquitination and proteasomal degradation^37^. In addition, IP3R are also substrates for the cysteine proteases, calpain and caspase^38^. Activation of either of these pathways following TNF receptor signaling could conceivably account for the reduction in IP3R levels. Future work is necessary to probe the detailed molecular signaling pathways responsible for the alteration in IP3R expression in the context of Sjögren’s Syndrome.

Generation of autoantibodies targeting functionally relevant proteins in the gland have been suggested as a major causal factor in pSS pathogenesis. In this context, anti-M3R autoantibodies have been identified in the sera from pSS patients. Since these antibodies directly target the M3R, one of the major neurotransmitter receptors involved in salivary fluid secretion, this autoantibody has received much attention. While there are some discrepancies in the reported findings^39^,^40^,^41^, it is feasible to hypothesize that if there were high levels of circulating anti-M3R *in vivo* in the vicinity of the receptors in the gland, it could dampen the cellular response to neurotransmitter stimulation. Additionally, antibodies to IP3R have also been detected in sera from primary Sjögren’s 17 of 35 (48.6%), secondary Sjögren’s 13 of 39 (33%), and rheumatoid arthritis 34 of 124 (27.4%)^42^. However, it is unclear whether autoantibodies can induce permanent effects on the acinar cells which would lead to dampening of their response to (CCh) *in vitro*, as seen in the present study. Furthermore, our observation that the decrease in response is also seen at high (CCh) argues against an effect of ambient antibodies that are associated with the tissue. Defects in AQP5 have also been previously reported, which include a decrease as well as mis-localization of the channel, both of which could result in decreased water secretion. There are also data which refute such alterations in the distribution of AQP5 in acinar cells of glands in pSS patients^43^,^44^. Irrespective of the change in AQP5, since AQP5 insertion into the apical plasma membrane as well as fluid secretion *per se* is critically dependent on CCh-stimulated Ca^2+^ mobilization, loss of muscarinic receptor function or defects in Ca^2+^ mobilization would adversely impact water secretion. As discussed above, we do not see an overall decrease, or mislocalization, of AQP5 in acinar cells from pSS patients. However, based on the decreased Ca^2+^ mobilization that we have observed in acini from pSS patients, we suggest that AQP5 trafficking to the apical membrane, and consequently water secretion, will be reduced in this tissue. Consistent with this we have seen an association between CCh-stimulated Ca^2+^ mobilization and volume reduction in salivary gland acinar cells from individual patients.

Relatively few studies have examined acinar cell function in salivary glands from pSS patients. A previous report compared cell volume changes in salivary gland cells from pSS and non-pSS patients (individuals presented with dry mouth but did not meet criteria for pSS) but did not evaluate function in healthy volunteers. Further, only response of cells to hypo-osmotic stress was assessed where exposure of cells to a hypo-osmotic medium triggers an increase in cell volume that is followed by regulatory volume decrease^45^. Such volume changes are non-physiological but can be used to assess the overall water permeability of cells. However, this assay does not provide insight regarding the physiological response of cells to a secretagogue. This previous study showed that pSS patient cells had reduced cell swelling as well as volume recovery compared to the non-pSS group. While the investigators attributed the change to disruption of AQP5 in an around the infiltrating areas, alterations in other factors including cytoskeletal changes, volume-regulated Ca^2+^ channels, Ca^2+^-activated K^+^ channels, and volume regulated Cl^-^ channels could contribute to the defect in regulated volume decrease^46^. Another previous study by Dawson et al. demonstrated a change in the sensitivity of cells to acetylcholine^47^ with attenuation of [Ca^2+^]_i_ increases and Ca^2+^-dependent ion channel activation at relatively low [agonist] in pSS cells compared to healthy patients. However, as shown by our data, Ca^2+^ signaling in salivary gland acini from FS = 1 patients was dampened across all concentrations of (CCh) tested. Notably, [Ca^2+^]_i_ signaling in cells from IL14α-TG glands, appeared to recover at higher (CCh). Further studies will be required to evaluate whether the agonist sensitivity of the glands in pSS decreases temporally following disease onset. The most novel and important finding in the present study is that salivary gland acinar cells from morphologically intact areas of the glands from SS patients demonstrate a loss of IP3R3 and a mislocalization of IP3R2. To our knowledge none of the previously reported studies have examined acinar IP3Rs in Sjögren’s Syndrome. Consistent with the findings in patients, acini from IL14α-TG mice show a decrease in IP3R2 (IP3R3 have not yet been assessed). Thus, we suggest that IP3R deficit in acinar cells is the underlying cause for the secretory dysfunction in pSS patients who have low levels of inflammation and minimal tissue destruction in the salivary glands. While we have not directly assessed the intracellular Ca^2+^ store content in salivary glands from pSS patients, our data demonstrate that the Ca^2+^ store content is similar in acini from WT and IL14α-TG mice. Further studies are required to assess the temporal sequence of events, namely whether mislocalization of the protein precedes the loss. Interestingly, mice lacking IP3R2 and IP3R3 display loss of lacrimal secretion and progressive inflammation in the eyes, as seen in pSS patients^48^.

In conclusion, we present here novel data that demonstrate a decrease and mislocalization of IP3Rs in minor salivary gland acinar cells from pSS patients. This defect in IP3Rs can account for the accompanying decrease in agonist-stimulated Ca^2+^ signaling and cell volume regulation, which together fully account the loss of saliva secretion. Our findings suggest that a link between LTα and loss of IP3R in pSS-associated secretory dysfunction. Previous studies reported by Ambrus and co-workers demonstrate that IFNα is not responsible for the early stages of Sjögren’s Syndrome, including salivary dysfunction, in the submandibular and lacrimal glands^11^. Additionally, no trials have yet demonstrated the benefit for blocking INFα in Sjögren’s Syndrome. Another interesting observation from these previous studies is that lymphocytes participate in the destruction of the salivary glands. Indeed, we have noted in the present study that tissue destruction was maximum in the area within and surrounding the infiltrate. On the other hand, increase in LTα is associated with early stages of the disease and likely mediates its effects via activating TNF receptors locally in salivary glands. However, further studies are needed to delineate the site and mechanism involved in generation of LTα locally within the gland as well as identifying the intracellular signaling events that lead to decrease in IP3R as well. Understanding of these mechanisms will identify new targets as well as the optimal time-frame for effective treatment of the exocrinopathy and secretory dysfunction associated with Sjögren’s Syndrome.

## Materials and Methods

### Study Approval

All studies were carried out in accordance with approved NIH guidelines. Human samples were obtained from NIH Institutional Review Board approved protocols (ClinicalTrials.gov Identifiers: NCT00001196 and NCT00001852). All human experiments including the use of tissue samples were performed after informed consent was obtained from subjects in the Sjögren’s Syndrome Clinic at the National Institute of Dental and Craniofacial Research (NIDCR) at the National Institute of Health (NIH) in Bethesda, MD.

### Patient selection

46 subjects (HV+ pSS) were included in this study. 24 of the biopsies were from lower labial minor salivary gland from pSS patients who fulfilled the American-European consensus group criteria^4^. Irrespective of their menopausal status, all Healthy Volunteers enrolled in this study responded negatively to the questionnaire for the presence of oral symptoms, as per the European American Criteria for the diagnosis of Sjögren’s Syndrome. Biopsies were evaluated and assigned focus score [FS, defined as an accumulation of at least 50 inflammatory cells per mm^2^ 49]. 5 patients had FS = 0, 11 had FS = 1, 2 had FS = 2, 3 had FS = 3, 2 had FS = 4, and 1 patient had FS = 5 (see details in Supplemental Table 1). 22 biopsies were collected from healthy volunteers, who underwent examination to confirm their health status, including subjective evaluation of ocular and oral symptoms (using questionnaire), schirmer’s test and positive vital dye staining, histopathology (minor salivary gland biopsy), saliva measurements, and presence of autoantibodies in serum.

### Preparation of cell lobules

The biopsy was placed in cold tyrode solution (in mM):130 NaCl, 5.4 KCl, 1CaCl_2,_ 10 HEPES, 1 MgCl_2_, 10 glucose, 1% BSA: pH 7.4, gassed with 95% O_2_5% CO_2_, finely minced and incubated with 1μM calcein-AM (Molecular Probes, Invitrogen, Eugene, OR, USA) or 10 μM Fluo2-AM (**TEF Labs Inc.** Austin, TX, U.S.A.) for 30 minutes. Cells were washed with tyrode solution for 30 minutes. The time frame between collection of the biopsy and performing live cell imaging experiments was an average of 2.5 hours. Lack of cell digestion provides a relatively intact *in situ* system to study salivary gland cell function^50^. In addition, the cell clusters were viable up to 6 to 8 hours. Lobules were isolated by dissection under a microscope from IL14α (TG) mice^12^ which are on a C57B6 background. They were prepared for imaging in an identical fashion to human biopies. Use of mice for the experiments described herein was approved by the University Committee on Animal Resources (UCAR), University of Rochester. The protocol number is UCAR-2001-214 and performed in accordance with the approved guidelines.

### Live cell imaging using confocal and multiphoton microscopy

Dye-loaded lobules were placed in a perfusion chamber on cover slips which were coated with cell tak (from BD Bioscience, Bedford, MA) in solution containing: 130 NaCl, 5.4 KCl, 1CaCl_2,_ 10 HEPES, 1 MgCl_2_, 10 glucose, pH 7.4. The clusters were imaged using a Leica SP2 confocal mounted on a DM IRE2 inverted microscope with 20 × (0.4 NA) dry objective and images were acquired every 1`s. Both Calcein and Fluo2 AM were excited with the 488-nm line of an argon ion laser with emission at 510 nm. In experiments employing multiphoton microscopy, Fluo2 loaded lobules were excited at 810 nm using a Spectra Physics tunable fS pulsed Ti-Sapphire laser controlled by Fluoview software on a Olympus FV1000MP microscope using a 25× water immersion objective (1.03 NA). The clusters were stimulated with 1 μm carbachol (CCh) (Sigma, St. Louis, MO, U.S.A.) and maintained at 37 ^°^C. Changes in Ca^2+^ in the medium are indicated in the text as is time of CCh addition. Images were acquired at a resolution of 512 × 512 pixels. Regions of interest were selected and fluorescence intensity in that region was determined as a function of time and expressed relative to the initial fluorescence.

### Immunofluorescence and histochemistry and H&E staining

Samples were deparaffinized, rehydrated followed by microwave antigen retrieval for 10 minutes in 1mM EDTA, pH 8.0 with 0.05% Tween 20. Sections were cooled and blocked with 10% donkey serum in PBS with 0.05% BSA for 30 minutes at room temperature, followed by incubation with primary antibody [rabbit anti-AQP5 (Santa Cruz biotech.), rabbit anti-STIM 1 (cell signaling) rabbit anti-IP3R3 (Novus) and anti-IP3R2^51^. Samples were washed and incubated with Alexa fluor 488-conjugated donkey anti-rabbit antibody at 1:100 (Molecular Probes), mounted and imaged using Olympus Fluoview 1000 using a 40X, 60x, or 100x objectives.

For H&E staining and morphology assessment, paraffin embedded human minor salivary gland sections were melted and then stained with Mayer’s Hematoxolin (Electron Microscopy Sciences) followed by Eosin–Phloxine B (Electron Microscopy Sciences), and washed. Sections were then mounted and cover slipped using Permount (Fisher) and images were acquired.

### Statistics

Data analysis was performed using Origin 9.0 (OriginLab) and PRISM 5. Statistical comparisons were made using Spearman r (95% confident Interval) and Student t test. P value Significant (alpha = 0.05). Experimental values are expressed as means +/− SEM. Differences in the mean values were considered to be significant at *p *= 0.05.

## Additional Information

**How to cite this article**: Teos, L. Y. *et al.* IP3R deficit underlies loss of salivary fluid secretion in Sjögren's Syndrome. *Sci. Rep.* **5**, 13953; doi: 10.1038/srep13953 (2015).

## Supplementary Material

Supplementary Information

## References

[b1] Delaleu N., Jonsson R. & Koller M. M. Sjogren’s syndrome. European journal of oral sciences 113, 101-113, 10.1111/j.1600-0722.2004.00183.x (2005). 15819815

[b2] Mavragani C. P. & Moutsopoulos H. M. The geoepidemiology of Sjogren’s syndrome. Autoimmunity reviews 9, A305–310, 10.1016/j.autrev.2009.11.004 (2010). 19903539

[b3] Fox R. I. Sjogren’s syndrome. Lancet 366, 321–331, 10.1016/S0140-6736(05)66990-5 (2005). 16039337

[b4] Vitali C. *et al.* Classification criteria for Sjogren’s syndrome: a revised version of the European criteria proposed by the American-European Consensus Group. Annals of the rheumatic diseases 61, 554–558 (2002). 12006334 10.1136/ard.61.6.554 PMC1754137

[b5] Hansen A., Lipsky P. E. & Dorner T. Immunopathogenesis of primary Sjogren’s syndrome: implications for disease management and therapy. Current opinion in rheumatology 17, 558–565 (2005). 16093833 10.1097/01.bor.0000172801.56744.c3

[b6] Nikolov N. P. & Illei G. G. Pathogenesis of Sjogren’s syndrome. Current opinion in rheumatology 21, 465–470, 10.1097/BOR.0b013e32832eba21 (2009). 19568172 PMC2766246

[b7] Mavragani C. P. & Moutsopoulos H. M. Sjogren’s Syndrome. Annual review of pathology 9, 273–285, 10.1146/annurev-pathol-012513-104728 (2014). 24050623

[b8] Fox R. I. & Stern M. Sjogren’s syndrome: mechanisms of pathogenesis involve interaction of immune and neurosecretory systems. Scandinavian journal of rheumatology. Supplement 116, 3–13 (2002). 12109541

[b9] Lee B. H., Gauna A. E., Pauley K. M., Park Y. J. & Cha S. Animal models in autoimmune diseases: lessons learned from mouse models for Sjogren’s syndrome. Clinical reviews in allergy & immunology 42, 35–44, 10.1007/s12016-011-8288-5 (2012). 22105703 PMC3712500

[b10] Shen L. *et al.* IL-14 alpha, the nexus for primary Sjogren’s disease in mice and humans. Clinical immunology 130, 304–312, 10.1016/j.clim.2008.10.006 (2009). 19038581

[b11] Shen L. *et al.* A role for lymphotoxin in primary Sjogren’s disease. J Immunol 185, 6355–6363, 10.4049/jimmunol.1001520 (2010). 20952683

[b12] Shen L. *et al.* Development of autoimmunity in IL-14 alpha-transgenic mice. J Immunol 177, 5676–5686 (2006). 17015757 10.4049/jimmunol.177.8.5676

[b13] Xuan J. *et al.* Temporal histological changes in lacrimal and major salivary glands in mouse models of Sjogren’s syndrome. BMC oral health 13, 51, 10.1186/1472-6831-13-51 (2013). 24093879 PMC4015998

[b14] Seleznik G. M., Zoller J., O’Connor T., Graf R. & Heikenwalder M. The role of lymphotoxin signaling in the development of autoimmune pancreatitis and associated secondary extra-pancreatic pathologies. Cytokine & growth factor reviews 25, 125–137, 10.1016/j.cytogfr.2014.01.003 (2014). 24508087

[b15] Ambudkar I. S. Dissection of calcium signaling events in exocrine secretion. Neurochemical research 36, 1212–1221, 10.1007/s11064-011-0465-7 (2011). 21534000 PMC3825030

[b16] Ambudkar I. S. Polarization of calcium signaling and fluid secretion in salivary gland cells. Current medicinal chemistry 19, 5774–5781 (2012). 23061636 10.2174/092986712804143321 PMC3840904

[b17] Melvin J. E., Yule D., Shuttleworth T. & Begenisich T. Regulation of fluid and electrolyte secretion in salivary gland acinar cells. Annual review of physiology 67, 445–469, 10.1146/annurev.physiol.67.041703.084745 (2005). 15709965

[b18] Berridge M. J. Inositol trisphosphate and calcium signalling. Nature 361, 315–325, 10.1038/361315a0 (1993). 8381210

[b19] Mikoshiba K. IP3 receptor/Ca^2+^ channel: from discovery to new signaling concepts. Journal of neurochemistry 102, 1426–1446, 10.1111/j.1471-4159.2007.04825.x (2007). 17697045

[b20] Singh B. B. *et al.* Trp1-dependent enhancement of salivary gland fluid secretion: role of store-operated calcium entry. FASEB journal: official publication of the Federation of American Societies for Experimental Biology 15, 1652–1654 (2001). 11427516 10.1096/fj.00-0749fje

[b21] Petersen O. H. Localization and regulation of Ca^2+^ entry and exit pathways in exocrine gland cells. Cell calcium 33, 337–344 (2003). 12765680 10.1016/s0143-4160(03)00047-2

[b22] Hong J. H. *et al.* Polarized but differential localization and recruitment of STIM1, Orai1 and TRPC channels in secretory cells. Traffic 12, 232–245, 10.1111/j.1600-0854.2010.01138.x (2011). 21054717 PMC3021582

[b23] Liu X. *et al.* Attenuation of store-operated Ca^2+^ current impairs salivary gland fluid secretion in TRPC1(−/−) mice. Proceedings of the National Academy of Sciences of the United States of America 104, 17542–17547, 10.1073/pnas.0701254104 (2007). 17956991 PMC2077292

[b24] Sugita M. *et al.* cAMP-Dependent potentiation of the Ca(2+)-activated release of the anionic fluorescent dye, calcein, from rat parotid acinar cells. European journal of pharmacology 388, 227–234 (2000). 10675730 10.1016/s0014-2999(99)00898-5

[b25] Sugita M., Shiba Y., Furuya K., Yamagishi S. & Kanno Y. Involvement of intracellular calcium ions in the release of the fluorescent dye calcein by cholinergic and alpha-adrenergic agonists from rat parotid acinar cells. Pflugers Archiv : European journal of physiology 429, 555–560 (1995). 7617446 10.1007/BF00704161

[b26] Ambudkar I. S. Ca signaling and regulation of fluid secretion in salivary gland acinar cells. Cell calcium, 10.1016/j.ceca.2014.02.009 (2014). PMC4059182 24646566

[b27] Futatsugi A. *et al.* IP3 receptor types 2 and 3 mediate exocrine secretion underlying energy metabolism. Science 309, 2232–2234, 10.1126/science.1114110 (2005). 16195467

[b28] Gresz V. *et al.* Identification and localization of aquaporin water channels in human salivary glands. American journal of physiology. Gastrointestinal and liver physiology 281, G247–254 (2001). 11408278 10.1152/ajpgi.2001.281.1.G247

[b29] Mikoshiba K. *et al.* The role of Ca2^+^ signaling in cell function with special reference to exocrine secretion. Cornea 27 Suppl 1, S3–8, 10.1097/ICO.0b013e31817f246e (2008). 18813072

[b30] Petersen O. H. & Tepikin A. V. Polarized calcium signaling in exocrine gland cells. Annual review of physiology 70, 273–299, 10.1146/annurev.physiol.70.113006.100618 (2008). 17850212

[b31] Yule D. I. Subtype-specific regulation of inositol 1,4,5-trisphosphate receptors: controlling calcium signals in time and space. The Journal of general physiology 117, 431–434 (2001). 11331353 10.1085/jgp.117.5.431 PMC2233654

[b32] Gottenberg J. E. *et al.* Activation of IFN pathways and plasmacytoid dendritic cell recruitment in target organs of primary Sjogren’s syndrome. Proceedings of the National Academy of Sciences of the United States of America 103, 2770–2775, 10.1073/pnas.0510837103 (2006). 16477017 PMC1413808

[b33] Shen L. *et al.* Different stages of primary Sjogren’s syndrome involving lymphotoxin and type 1 IFN. J Immunol 191, 608–613, 10.4049/jimmunol.1203440 (2013). 23772034

[b34] Wajant H., Pfizenmaier K. & Scheurich P. Tumor necrosis factor signaling. Cell death and differentiation 10, 45–65, 10.1038/sj.cdd.4401189 (2003). 12655295

[b35] Ivanova H. *et al.* Inositol 1,4,5-trisphosphate receptor-isoform diversity in cell death and survival. Biochimica et biophysica acta 1843, 2164–2183, 10.1016/j.bbamcr.2014.03.007 (2014). 24642269

[b36] Vanderheyden V. *et al.* Regulation of inositol 1,4,5-trisphosphate-induced Ca2^+^ release by reversible phosphorylation and dephosphorylation. Biochimica et biophysica acta 1793, 959–970, 10.1016/j.bbamcr.2008.12.003 (2009). 19133301 PMC2693466

[b37] Wojcikiewicz R. J., Ernst S. A. & Yule D. I. Secretagogues cause ubiquitination and down-regulation of inositol 1, 4,5-trisphosphate receptors in rat pancreatic acinar cells. Gastroenterology 116, 1194–1201 (1999). 10220512 10.1016/s0016-5085(99)70023-5

[b38] Alzayady K. J., Chandrasekhar R. & Yule D. I. Fragmented inositol 1,4,5-trisphosphate receptors retain tetrameric architecture and form functional Ca2^+^ release channels. The Journal of biological chemistry 288, 11122–11134, 10.1074/jbc.M113.453241 (2013). 23479737 PMC3630841

[b39] Cavill D., Waterman S. A. & Gordon T. P. Failure to detect antibodies to extracellular loop peptides of the muscarinic M3 receptor in primary Sjogren’s syndrome. The Journal of rheumatology 29, 1342–1344 (2002). 12064859

[b40] Dawson L. J. *et al.* Antimuscarinic antibodies in primary Sjogren’s syndrome reversibly inhibit the mechanism of fluid secretion by human submandibular salivary acinar cells. Arthritis and rheumatism 54, 1165–1173, 10.1002/art.21764 (2006). 16572451

[b41] Roescher N., Kingman A., Shirota Y., Chiorini J. A. & Illei G. G. Peptide-based ELISAs are not sensitive and specific enough to detect muscarinic receptor type 3 autoantibodies in serum from patients with Sjogren’s syndrome. Annals of the rheumatic diseases 70, 235–236, 10.1136/ard.2010.129049 (2011). 20498204 PMC6234417

[b42] Miyachi K. *et al.* Inositol 1,4,5-trisphosphate receptors are autoantibody target antigens in patients with Sjogren’s syndrome and other systemic rheumatic diseases. Modern rheumatology/the Japan Rheumatism Association 17, 137–143, 10.1007/s10165-006-0555-6 (2007). 17437169

[b43] Beroukas D., Hiscock J., Jonsson R., Waterman S. A. & Gordon T. P. Subcellular distribution of aquaporin 5 in salivary glands in primary Sjogren’s syndrome. Lancet 358, 1875–1876, 10.1016/S0140-6736(01)06900-8 (2001). 11741631

[b44] Gresz V., Horvath A., Gera I., Nielsen S. & Zelles T. Immunolocalization of AQP5 in resting and stimulated normal labial glands and in Sjogren’s syndrome. Oral diseases. 10.1111/odi.12239 (2014). 24661359

[b45] Enger T. B., Aure M. H., Jensen J. L. & Galtung H. K. Calcium signaling and cell volume regulation are altered in Sjogren’s Syndrome. Acta odontologica Scandinavica 72, 549–556, 10.3109/00016357.2013.879995 (2014). 24471729

[b46] Liu X. *et al.* A role for AQP5 in activation of TRPV4 by hypotonicity: concerted involvement of AQP5 and TRPV4 in regulation of cell volume recovery. The Journal of biological chemistry 281, 15485–15495, 10.1074/jbc.M600549200 (2006). 16571723

[b47] Dawson L. J., Field E. A., Harmer A. R. & Smith P. M. Acetylcholine-evoked calcium mobilization and ion channel activation in human labial gland acinar cells from patients with primary Sjogren’s syndrome. Clinical and experimental immunology 124, 480–485 (2001). 11472412 10.1046/j.1365-2249.2001.01526.x PMC1906076

[b48] Inaba T. *et al.* Mice lacking inositol 1,4,5-trisphosphate receptors exhibit dry eye. PloS one 9, e99205, 10.1371/journal.pone.0099205 (2014). 24901844 PMC4047094

[b49] Vitali C., Moutsopoulos H. M. & Bombardieri S. The European Community Study Group on diagnostic criteria for Sjogren’s syndrome. Sensitivity and specificity of tests for ocular and oral involvement in Sjogren’s syndrome. Annals of the rheumatic diseases 53, 637–647 (1994). 7979575 10.1136/ard.53.10.637 PMC1005429

[b50] Warner J. D. *et al.* Visualizing form and function in organotypic slices of the adult mouse parotid gland. American journal of physiology. Gastrointestinal and liver physiology 295, G629–640, 10.1152/ajpgi.90217.2008 (2008). 18669626 PMC2536791

[b51] Betzenhauser M. J. *et al.* ATP modulation of Ca2^+^ release by type-2 and type-3 inositol (1, 4, 5)-triphosphate receptors. Differing ATP sensitivities and molecular determinants of action. The Journal of biological chemistry 283, 21579–21587, 10.1074/jbc.M801680200 (2008). 18505727 PMC2490775

