# Supplementary material for: IP3R deficit underlies loss of salivary fluid secretion in Sjögren’s Syndrome
Source: Sci Rep. 2015 Sep 14;5:13953. doi: 10.1038/srep13953 (PMC4568516; doi:10.1038/srep13953)

IP3R deficit underlies loss of salivary fluid secretion in Sjögren's Syndrome

Leyla Y. Teos [1,2], Yu Zhang [3#], Ana P. Cotrim[1#], William Swaim [2] Jon H. Won [3], Julian Ambrus [4], Long Shen[4], Lolita Bebris [1], Margaret Grisius [1], Shyn-Ing Jang[1], David Yule [3], Indu Ambudkar [2]*, Ilias Alevizos [1]*

Supplemental Information

LEGEND

Table 1: Clinical features of all subjects

All twenty–four patients fulfilled the American-European consensus group criteria. There were twenty-two biopsies from healthy volunteers, and these patients underwent a full examination determining them as healthy volunteers. The criteria used for determining decreased salivary flow are ≤ 1.5 ml/15 minutes. This value is taken from the whole unstimulated salivary flow measurement and used for criteria purposes. Positive autoantibodies were determined by the presence of anti-Ro/SSA, anti-La/SSB antibodies or anti-nuclear antibodies.

SUPPLEMENTAL FIGURE LEGENDS

Supplemental Figure 1: Patient saliva flow and Ca^{2+} release and influx correlate.

Correlation graph displaying salivary flow and calcium release (Spearman r = 0.5741) (*p* = 0.0127) and influx (Spearman r = 0.5844) (*p* = 0.0109). Number of XY pairs 18 (HV = 7, pSS patients = 11).

Supplemental Figure 2: Stitched Immunofluorescence images of STIM1 & AQP5, respectively, in entire biopsied sample area. Representative image of a pSS patient with a FS of 1 & 3 and HV of STIM1 and AQP5. Regions marked IF = near and surrounding infiltration site and N = normal appearing tissue for pSS patients. Chosen regions of interest for HV regions

were chosen random area # 1-2 which corresponds to the enlarged images shown supplemental figure 3 STIM1, and Supplemental Figure 4 for AQP5.(Bar graph = 300 μM).

Supplemental Figure 3: STIM1 expression in minor salivary glands biopsies of pSS patients and healthy volunteers. Representative images of STIM1 detected by immunofluorescence in salivary gland sections from pSS patients with FS=1, FS=3, and healthy volunteers (HV) (scale bar = 50 microns). Areas were picked from stitched images as shown in Supplemental Fig 2. An area from within the infiltration (IF) was picked and an area away from infiltration where tissue appeared to be morphologically intact (N). In each case, enlarged images (of areas marked by white boxes) are shown in the second panel to the right (scale bar = 20).

Supplemental Figure 4: AQP5 expression in minor salivary glands biopsies of pSS patients and healthy volunteers. Representative images of AQP5 detected by immunofluorescence in salivary gland sections from pSS patients with FS=1, FS=3, and healthy volunteers (HV) (scale bar = 50 microns). Areas were picked from stitched images as shown in Supplemental Fig 2. An area from within the infiltration (IF) was picked and an area away from infiltration where tissue appeared to be morphologically intact (N). In each case, enlarged images (of areas marked by white boxes) are shown in the second panel to the right (scale bar = 20microns).

Supplemental Figure 5: Ca^{2+} store content is not altered in acinar cells from IL14 alpha transgenic mice. Cyclopiazonic acid**,** CPA, treatment in Ca-free external medium was used to assess intracellular Ca^{2+}-store content of salivary gland acinar cells in the two sets of mice. Lobule preparations were made as described above and samples were treated with CPA. **(**A)

Representative traces from female, age matched wild type controls showing changes in $[Ca^{2+}]_i$ stimulated by CPA submandibular acini. The traces represent the normalized change in fluorescence for an individual cell within an acinus**.** The data shown in A were used to calculate peak height (**B**), time to peak (**C**) and area under the curve (**D**). The data were obtained from 4 wild type mice in which 14 lobules were analyzed (8-10 months old) and 3 IL14a transgenic mice with 13 lobules (8-10 month old). There were no significant differences between the two groups in B-D.

Table 1: Clinical features of all subjects involved in the study

Subject_Number	Gender	Diagnosis	Visit_Age	Anti-Nuclear ABS Screen EU	Anti-Sjögren's SY.A EU	Anti- Sjögren's SY.B EU	Whole Unstimulated Salivary Flow	Focus Score
1	Female	Primary	26	10.7	238	104	0.5901ml/15mins	0
2	Female	Primary	29	8.9	237	NEG	8.1039ml/15mins	0
3	Female	Primary	36	>12.0	>300	92	3.8184ml/15mins	3
4	Female	Primary	38	NEG	NEG	NEG	1.7613ml/15mins	1
5	Female	Primary	39	NEG	NEG	NEG	1.1727ml/15mins	5
6	Female	Primary	41	10.8	>300	NEG	0ml/15mins	4
7	Female	Primary	44	4.4	NEG	31	6.702ml/15mins	1
8	Female	Primary	45	1.6	NEG	81	0ml/15mins	0
9	Female	Primary	49	5.6	132	NEG	0.0825ml/15mins	1
10	Female	Primary	49	>12.0	NEG	NEG	1.6671ml/15mins	1
11	Female	Primary	50	11.6	257	NEG	2.7759ml/15mins	1
12	Female	Primary	51	NEG	NEG	NEG	1.8351ml/15mins	1
13	Female	Primary	52	NEG	NEG	NEG	0.7086ml/15mins	1
14	Female	Primary	54	NEG	38	NEG	2.0574ml/15mins	1
15	Female	Primary	55	1.1	NEG	NEG	0.6816ml/15mins	1
16	Female	Primary	57	NEG	NEG	NEG	2.9454ml/15mins	1
17	Female	Primary	59	8.8	>300	NEG	0ml/15mins	4
18	Female	Primary	60	1.1	45	NEG	0.1041ml/15mins	0
19	Female	Primary	61	1.4	NEG	64	0.0726ml/15mins	0
20	Female	Primary	62	3.7	155	NEG	0.318ml/15mins	2
21	Female	Primary	64	6.3	164	NEG	1.0866ml/15mins	3
22	Female	Primary	70	9.4	166	90	0.2634ml/15mins	2
23	Female	Primary	70	1.7	NEG	NEG	4.131ml/15mins	1
24	Female	Primary	70	2.2	87	36	1.3413ml/15mins	3
1	Female	HV	21	NEG	NEG	NEG	3.8553ml/15mins	—
2	Female	HV	23	NEG	NEG	NEG	6.5901ml/15mins	—
3	Female	HV	24	NEG	NEG	NEG	6.1836ml/15mins	—
4	Female	HV	24	NEG	NEG	NEG	6.9699ml/15mins	—
5	Female	HV	24	NEG	NEG	NEG	3.9573ml/15mins	—
6	Female	HV	25	NEG	NEG	NEG	11.0649ml/15mins	—
7	Female	HV	26	NEG	NEG	NEG	1.8942ml/15mins	—
8	Female	HV	26	NEG	NEG	NEG	4.3236ml/15mins	—
9	Female	HV	27	NEG	NEG	NEG	10.5888ml/15mins	—
10	Female	HV	29	NEG	NEG	NEG	11.8074ml/15mins	—
11	Female	HV	30	NEG	NEG	NEG	16.4337ml/15mins	—
12	Female	HV	32	2.7	NEG	NEG	2.9271ml/15mins	—
13	Female	HV	35	NEG	NEG	NEG	8.1495ml/15mins	—
14	Female	HV	37	NEG	NEG	NEG	2.241ml/15mins	—
15	Female	HV	41	1.6	NEG	NEG	3.8052ml/15mins	—
16	Female	HV	47	NEG	NEG	NEG	9.3831ml/15mins	—
17	Female	HV	49	NEG	NEG	NEG	4.2984ml/15mins	—
18	Female	HV	51	NEG	NEG	NEG	1.5738ml/15mins	—
19	Female	HV	51	NEG	NEG	NEG	3.9474ml/15mins	—
20	Female	HV	51	7.3	NEG	NEG	2.4462ml/15mins	—
21	Male	HV	55	NEG	NEG	NEG	5.7639ml/15mins	—
22	Male	HV	55	NEG	NEG	NEG	0ml/15mins	—

Supplemental Figure 1

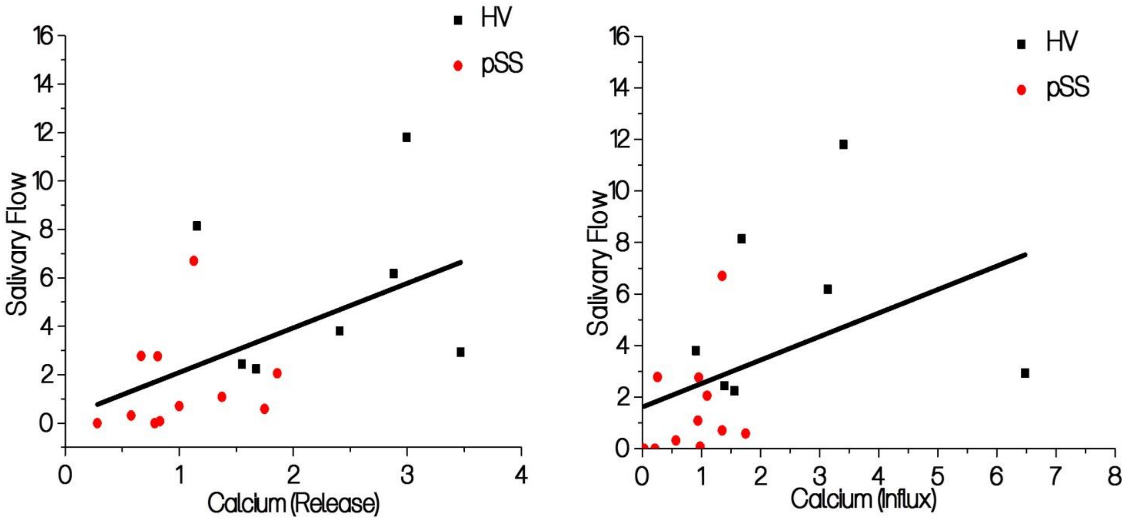

Supplemental Figure 2

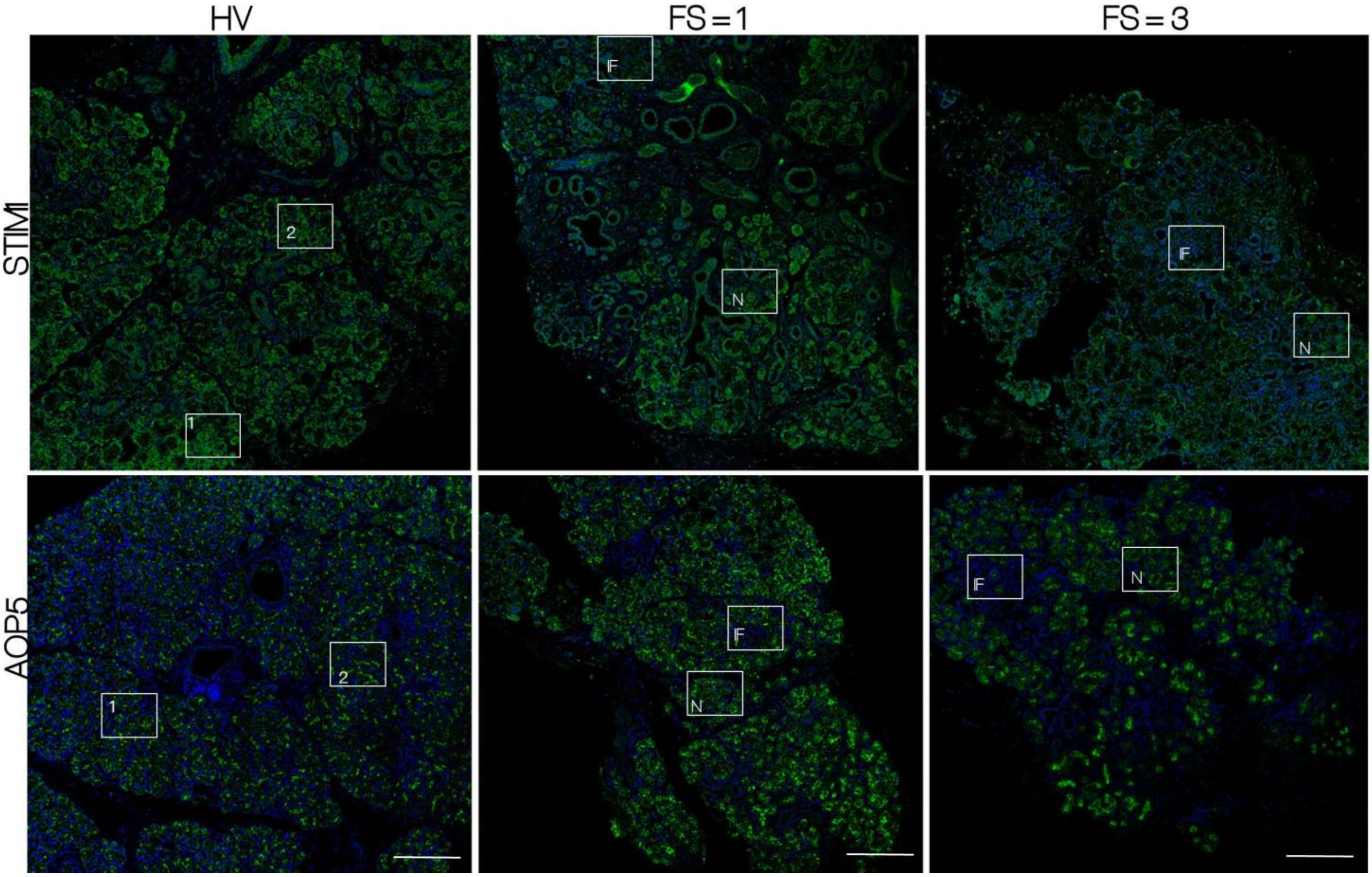

Supplemental Figure 3

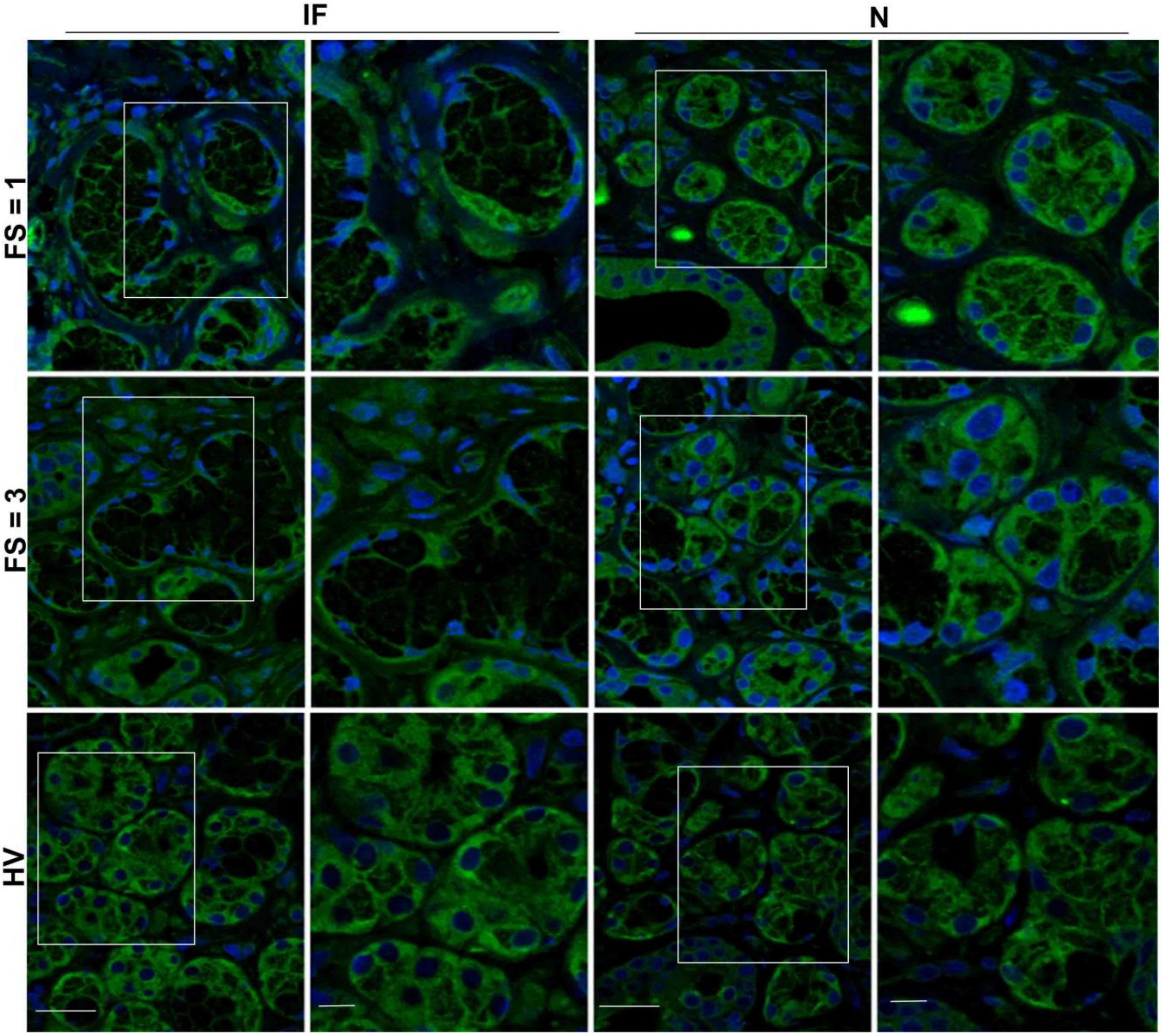

Supplemental Figure 4

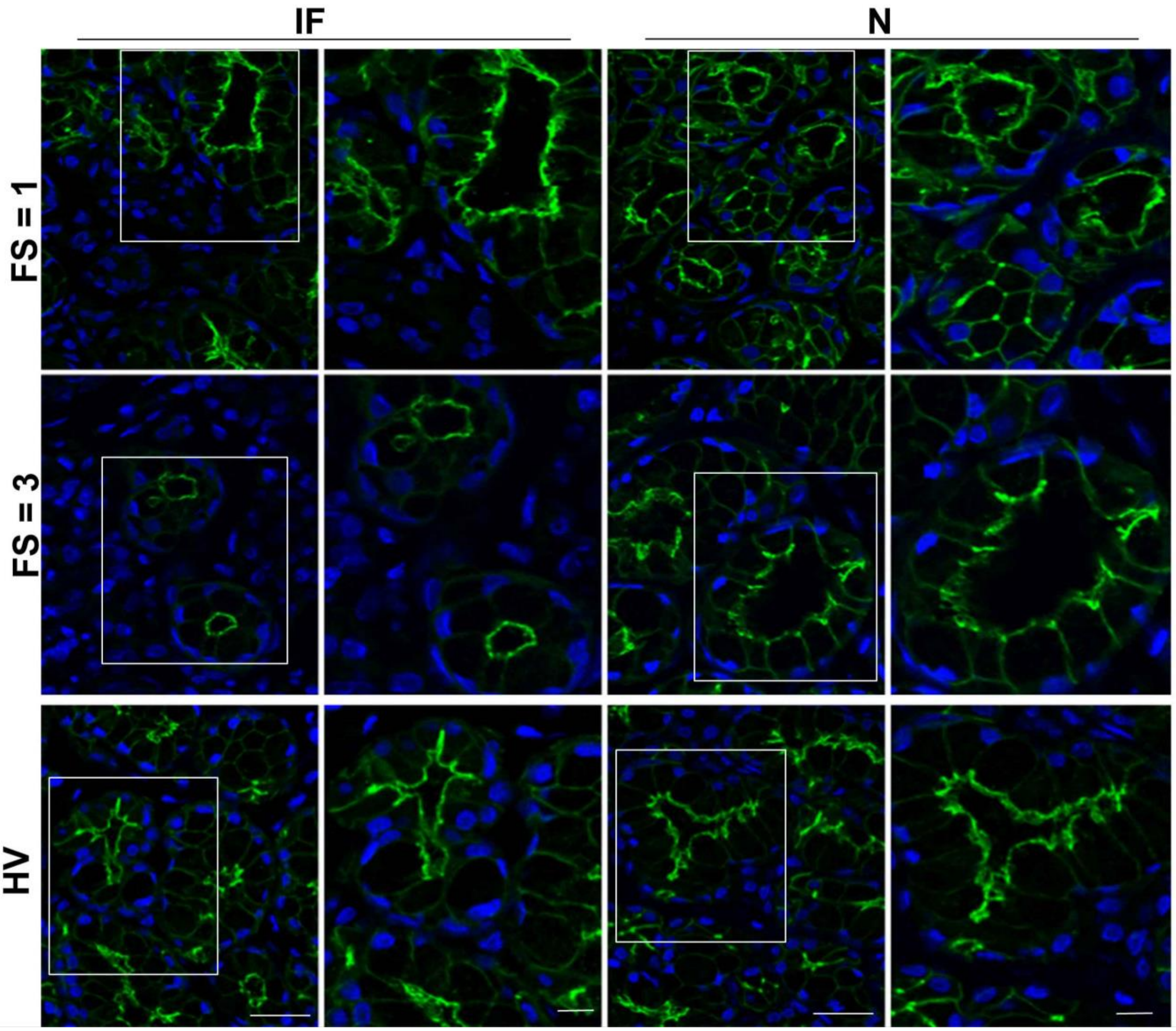

Supplemental Figure 5

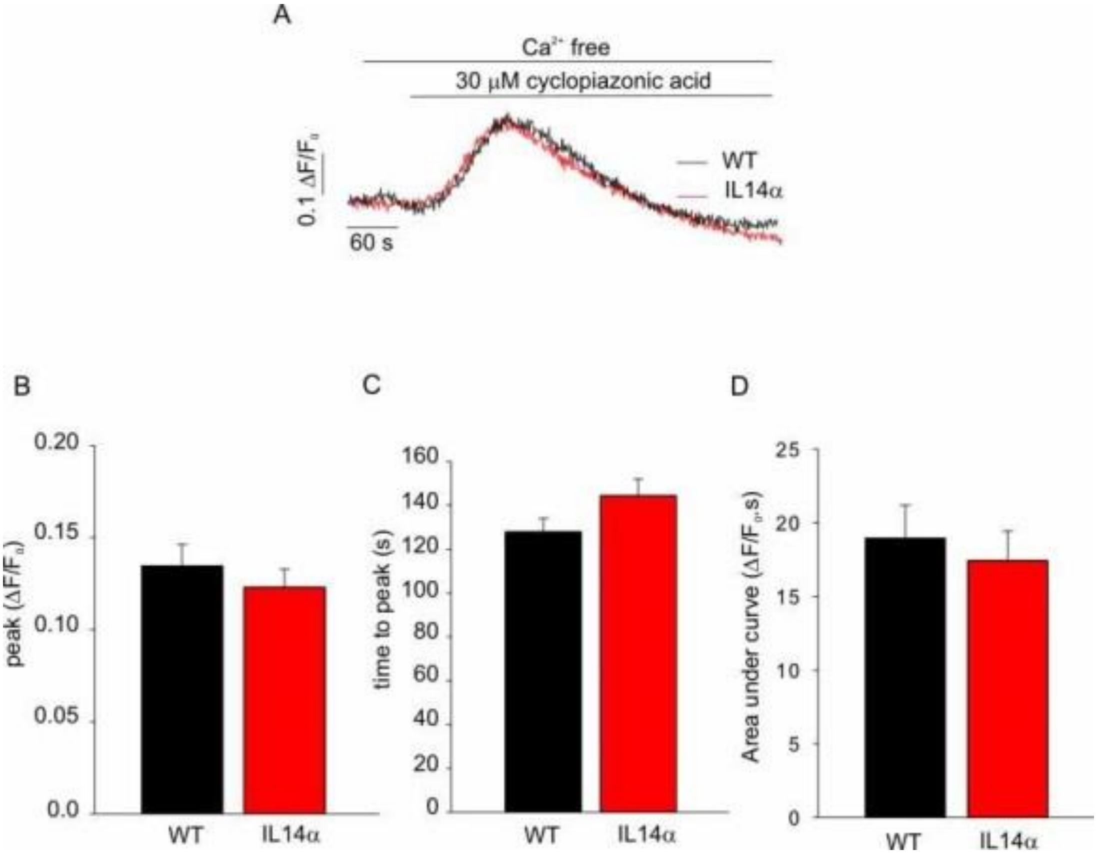

Supplement: Supplementary Information [file srep13953-s1.pdf]
